# The sensory feedback mechanisms enabling couples to walk synchronously: An initial investigation

**DOI:** 10.1186/1743-0003-4-28

**Published:** 2007-08-08

**Authors:** Ari Z Zivotofsky, Jeffrey M Hausdorff

**Affiliations:** 1 Gonda Brain Research Center, Bar Ilan University, Ramat Gan, Israel; 2 Laboratory for Gait & Neurodynamics, Movement Disorders Unit, Tel-Aviv Sourasky Medical Center, Tel-Aviv, Israel; 3 Department of Medicine, Harvard Medical School, Boston, MA, USA; 4 Physical Therapy Department; Sackler School of Medicine, Tel Aviv, Israel

## Abstract

The inattentive eye often will not notice it, but synchronization among human walking partners is quite common. In this first investigation of this phenomenon, we studied its frequency and the mechanisms that contribute to this form of "entrainment." Specifically, by modifying the available communication links between two walking partners, we isolated the feedback mechanisms that enable couples to synchronize their stepping pattern when they walk side-by-side. Although subjects were unaware of the research aims and were not specifically asked to walk in synchrony, we observed synchronized walking in almost 50% of the walking trials, among couples who do not usually walk together. The strongest in-phase synchrony occurred in the presence of tactile feedback (i.e., handholding), perhaps because of lower and upper extremity coupling driven in part by arm swing. Interestingly, however, even in the absence of visual or auditory communication, couples also frequently walked in synchrony while 180 degrees out-of-phase, likely using different feedback mechanisms. These findings may partially explain how patients with certain gait disorders and disturbed rhythm enhance their gait when they walk with a partner and suggest alternative interventions that might improve the stepping pattern. Further, this preliminary investigation highlights the relatively ubiquitous nature of an interesting phenomenon that has not previously been studied and suggests that further work is needed to better understand the mechanisms that entrain the gait of two walking partners and allows couples to walk in synchrony with minimal or no conscious effort.

## Background

**Figure 1:**
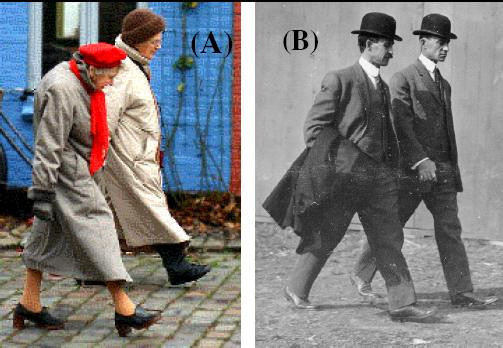
Example of two couples walking in synchrony. (A) Two older adults walking in the street and (B) the Wright brothers at the Belmont International Airmeet. The walking partners do not appear to be looking at each other and there is no apparent contact, yet the partners apparently walk as one. In the present study, we investigate the factors that enable synchronized walking such as that seen in these two examples.

When two people walk together, they often appear to step in almost perfect synchrony (e.g., see Fig. 1a and 1b). Step length and step time can be varied in numerous ways to ensure that walking partners move forward at the same rate and arrive at the desired destination together. However, instead of achieving this goal through a random, time-varying combination of these parameters, remarkably, couples often seem to march to a single drummer. What sensory mechanisms are employed to synchronize their walking patterns?

This phenomenon of independent organisms working in synchrony is not unique to people walking together. A wide variety of physical and physiological phenomena synchronize their actions [1]. This entrainment of independent entities generally requires a form of communication. Isolated heart cells beat synchronously in a properly adjusted cell culture, and individuals in an audience clap in unison using auditory feedback. More subtle forms of communication synchronize the menstrual cycle of women who live together in close proximity [2] and enable locusts to couple the beating of their wings [3]. For example, the rhythmically oscillating airflow from the front locust's wing-beat transmits the timing information needed for synchronization to the rear individual [3]. While these examples are not gait related, they represent instances where separate individuals synchronize a biological facet via a specific feedback mechanism. It is this sort of synchrony and feedback that we are investigating in this study. Certain patients with severe gait disturbances may improve their stepping pattern when they walk with a partner [4], and anecdotally, we have observed that these patients often entrain their walking pattern to that of their partner's, using synchronization to generate near-normal walking. However, the mechanisms that enable this entrainment of the walking pattern are largely unknown.

The gait of healthy adults is generally considered to be an automated motor task. In fact, walking is actually a highly complex, hierarchical process that is regulated by multiple internal brain networks and feedback mechanisms. Patients with disturbances in these mechanisms (e.g., Parkinson's disease) may use external sources such as periodic visual or auditory stimuli to synchronize their gait and thereby improve their walking rhythm [5,6]. What sort of communication is utilized when a couple walks together in synchrony? Visual, auditory, and tactile feedback are likely candidates. We tested this idea by isolating each of these factors and quantifying the degree of synchrony as couples walked side-by-side. Although this phenomenon may be observed in everyday experience, to our knowledge, this is the first attempt to examine its frequency and study the sensory feedback and coupling mechanisms that contribute to synchronized walking when couples walk side-by-side. Specifically, we tested the hypothesis that visual, auditory, and tactile communication between walking partners enables couples to walk in synchrony.

## Methods

Twenty-eight (14 pairs) middle school (13.8 ± 1.1 yrs), healthy young women were studied. Parental approval was provided and all provided informed consent prior to the study and all were blinded to the objectives of the study. Each pair walked under four different conditions that modified the available feedback. In each condition, participants were told to walk from one end of a 15-meter long hallway to the other end when they received the "start" signal. No instructions were given regarding "synchrony" and this idea was never explicitly mentioned to the participants. Please note that their starting simultaneously does not necessarily dictate synchrony: Their very first steps could have been of different stride lengths or step rates. Feedback, specifically with respect to the walking partner, was modified using side-blinders to decrease the visual field and prevent subjects from seeing their partner (no Visual Feedback condition), using headphones though which white noise was played to prevent subjects from hearing their partner's steps (no Auditory Feedback condition) and/or by having subjects walk while holding hands (Tactile Feedback condition). Thus, the four feedback conditions were:

1. Auditory feedback was unaltered. No visual or tactile feedback.

2. Visual feedback was unaltered. No auditory or tactile feedback.

3. Tactile feedback provided via handholding. No auditory or visual feedback.

4. No feedback (i.e., no auditory, visual or tactile feedback).

During each trial, subjects walked side-by-side on level ground in a quiet, obstacle free, well-lit hallway. They were asked to walk from one end of the hallway to the other end (about 15 m) at their normal pace. The lower extremities of the subjects were videotaped and two experts in gait analysis (physical therapists) reviewed the videos offline. The reviewers were told to focus on the middle 1/3 of each walk in order to study the steady-state behavior. Reviewers assigned to each trial a score from -3 to 3; 0 reflects total absence of synchronization, 3 reflects good (e.g., consistent) in-phase synchronization (e.g., left leg of one partner moved in time with the left leg of the other partner), and -3 reflects good (e.g., consistent) 180 degrees out-of-phase synchronization (e.g., left leg of one partner moved with the right leg of the other partner). Scores of 1.0 and 2.0 reflect mild and moderate in-phase synchronization, respectively. Each review was performed without knowledge of the results of the other and without knowledge of the specific study questions. There was good agreement between the two reviewers (Intraclass Correlation Coefficient, ICC, alpha = 0.87). Scores were averaged across the reviewers. To determine if the frequency of synchronization was different from chance, binomial tests were performed to test the hypothesis that the proportion of couples demonstrating synchronization (a binary variable that was positive if the average absolute scores were greater than 1.0) in any given feedback condition was statistically different from 0.1 (i.e., 10%). To test whether the degree of synchronization was different from the no feedback condition, paired t-tests were performed comparing this condition to the others.

## Results

**Figure 2:**
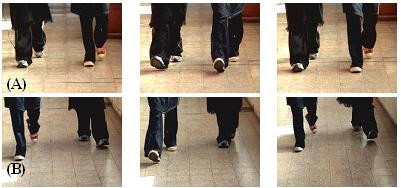
(A) Snapshots taken during a *tactile feedback *(handholding) trial that received a score of 3.0 (good in-phase synchrony). Note how the heel-strike and toe-off of both walking partners occur simultaneously. (B) Snapshots of the same couple shown in (A) during a *no feedback *trial that received a score of -0.5 (poor synchrony). A suggestion of out-of-phase synchrony can be observed in the left and right panels, but this is not consistent (see middle panel).

**Figure 3:**
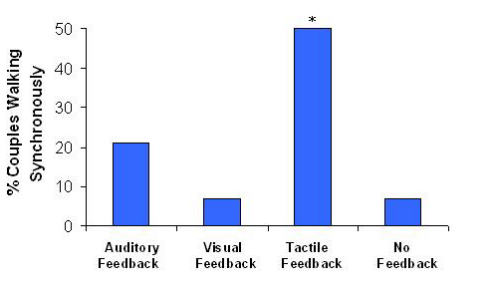
Frequency of in-phase synchronized walking (synchrony score>1.0) as a function of the specific type of feedback. In the tactile feedback condition (handholding without visual or auditory feedback), 50% of the couples walked in synchrony in-phase. * Indicates significant difference from chance, (i.e., > 10% in good synchrony, p < 0.001).

Synchronized walking (absolute value of synchrony score> 1.0) occurred frequently (26 out of 56 trials, 46%). Among all conditions, in-phase synchrony (score> 1.0) occurred most frequently (50% of trials in this condition) with tactile feedback (see Fig. 2a and Fig. 3). The synchrony score in the no feedback condition (see, for example, Fig. 2b) was significantly lower than that of the tactile condition (p < 0.05), but was not different from the auditory or visual feedback conditions (p > 0.32). Handholding apparently promotes in-phase synchrony, but visual and auditory feedback apparently do not.

A different, more puzzling picture is observed for out-of-phase synchrony. Among the 56 walking trials, the frequency of this type of synchrony was similar to that of in-phase synchrony (14/56 vs. 12/56, respectively). For this type of coupled walking, the frequency of good synchrony and the synchronization scores were similar in the auditory, tactile and no feedback conditions (29%, 29%, and 36%, respectively). In contrast, walking with intact visual feedback had a negative effect on synchrony, reducing the observed frequency (to 7%, i.e., only 1 couple) and lowering the magnitude (p = 0.014, compared to the no feedback condition). Differences in height among the walking partners did not explain when out-of-phase synchrony occurred instead of in-phase walking, and were not associated with the presence or absence of any kind of synchrony (p > 0.15).

## Discussion

In this first study of synchronized walking, we found that its frequency is relatively high. Although the couples were never asked to walk synchronously and this was the first time they walked together as couples, they did in fact walk as a synchronized unit in almost 50% of all the trials. This is far above what might be expected by chance; there are an infinite number of combinations of stride length and step rates that can produce a given walking speed, and left to chance, two walkers should essentially never synchronize. The interesting finding is those conditions in which there is any synchrony, not those with low probabilities of synchrony. These results confirm our anecdotal observations; having become sensitive to the possibility that couples may walk in synchrony, we observe that two walking partners are often unconsciously walking in synchrony. A natural question addressed here is what contributes to the ubiquity of this phenomenon. This preliminary investigation revealed that in the presence of tactile feedback, in-phase synchrony is likely while neither visual nor auditory feedback increase the likelihood of in-phase synchrony above that seen with no feedback. It may be that vision acts as a distraction, thus interfering with the ability of the walkers to synchronize.

The mechanisms for the first part of these results may be explainable on the basis of previous investigations that demonstrated that the light touch of a single finger markedly improves balance and reduces postural sway [7-9]. Here we demonstrate the importance of light touch on step timing. Synchronized walked occurred when couples held hands, while visual and auditory feedback between the walking partners was blocked. One possibility is that the light touch of handholding provides a "communication link" that synchronizes the two walking partners. Alternatively, one could argue that this finding is anticipated. Hand-holding also imposes a biomechanical constraint, to some degree, and within individuals, arm swing and step timing may be interconnected [10,11]. Thus, perhaps the stepping pattern of one subject determines the movement of the arm in that subject, which in turn, sets the arm movement pattern and then the step timing of the walking partner. In other words, perhaps, lower limb/upper limb coupling of one individual dictates arm swing timing, which in turn determines the arm swing timing, and hence, lower limb pattern of the partner. Nonetheless, despite this constraint, it is important to note that step length and step time can be combined in many ways, even in the presence of hand-holding, without necessarily imposing synchrony. Indeed, in the present study, half of the couples walked in synchrony in this condition while half did not. It remains to be determined if hand-holding predisposes to synchronized walking by providing tactile feedback, perhaps similar to light-touch, or if the biomechanical constraint is the key factor. Clearly, however, handholding alone is not strong enough to evoke unconscious synchronized walking one hundred percent of the time. Understanding the role of tactile feedback in healthy subjects may suggest how touch and hand holding helps patients with certain types of gait disturbances because they appear to utilize similar mechanisms when they walk with a partner.

The present findings suggest that synchronized walking often occurs even if in the absence of tactile feedback or biomechanical constraints. In fact, synchronized walking (in and out of phase) was observed in many trials in which subjects did not hold hands. Biomechanical constraints cannot explain the occurrence of synchronized walking in these cases. An alternative, perhaps complimentary, explanation for the observed results relates to a recent investigation [12] of six pairs of subjects who walked towards or away from one another, with one subject tending to "follow" or imitate the gait pattern of the other. The authors suggest that bidirectional interactions with mutual influences of each subject on one another are responsible for this behavior. While vision tended to have a negative effect in the present study, minimizing the importance of "imitation", perhaps similar bidirectional mechanisms may be influencing the gait of subjects who walk side-by-side.

In the future, it might be helpful to compare side-by-side walking with walking as subjects approach or move away from each other, and to quantify the kinematics and kinetics of the walking partners using video markers, EMG, and/or footswitches, methods that are more quantitative than the method used in the present study. The qualitative assessment used was a limitation of the present study. In addition, to better understand the role of attention and the cognitive function required to walk in synchrony, it might also be informative to examine the effects of dual tasking on side-by-side synchrony. Further, in the present study, subjects were assessed during the steady state portion of a 15 meter walk. It might be helpful to evaluate "start-up" effects as subjects reach a steady state and to study synchronization during a longer walk.

This initial investigation suggests that synchronized walking is relatively commonplace and that it is apparently an example of a more general phenomenon in which complex biological rhythms become synchronized via readily apparent as well as more inconspicuous mechanisms. Identification of all of the sources of this synchronization may be used to better understand the mechanisms behind a perplexing everyday phenomenon and perhaps, ultimately, to enhance the walking pattern of patients with disturbed gait rhythm.

## Competing interests

The author(s) declare that they have no competing interests.

## Authors' contributions

AZZ designed the study, supervised data collection, and drafted the manuscript. JMH participated in the design of the study, helped with statistical analyses, and edited the manuscript. Both authors read and approved the final manuscript.

## References

[B1] Glass L (2001). Synchronization and rhythmic processes in physiology. Nature.

[B2] Stern K, McClintock MK (1998). Regulation of ovulation by human pheromones. Nature.

[B3] Camhi J, Sumbre G, Wendler G (1995). Wing-beat coupling between flying locust pairs: preferred phase and lift enhancement. J Exp Biol.

[B4] Hadar-Frumer M, Giladi N, Hausdorff JM (2004). Idiopathic "cautious" gait disorder of the elderly: Effects of reducing fear of failing. Movement Disorders.

[B5] Morris ME, Iansek R, Matyas TA, Summers JJ (1996). Stride length regulation in Parkinson's disease. Normalization strategies and underlying mechanisms. Brain.

[B6] Rubenstein TC, Giladi N, Hausdorff JM (2002). The power of cueing to circumvent dopamine deficits: A review of physical therapy treatment of gait disturbances in Parkinson's disease. Mov Disord.

[B7] Dickstein R, Shupert CL, Horak FB (2001). Fingertip touch improves postural stability in patients with peripheral neuropathy. Gait Posture.

[B8] Dickstein R, Peterka RJ, Horak FB (2003). Effects of light fingertip touch on postural responses in subjects with diabetic neuropathy. J Neurol Neurosurg Psychiatry.

[B9] Lackner JR, Rabin E, DiZio P (2001). Stabilization of posture by precision touch of the index finger with rigid and flexible filaments. Exp Brain Res.

[B10] Kubo M, Wagenaar RC, Saltzman E, Holt KG (2004). Biomechanical mechanism for transitions in phase and frequency of arm and leg swing during walking. Biol Cybern.

[B11] Wagenaar RC, van Emmerik RE (2000). Resonant frequencies of arms and legs identify different walking patterns. J Biomech.

[B12] Ducourant T, Vieilledent S, Kerlirzin Y, Berthoz A (2005). Timing and distance characteristics of interpersonal coordination during locomotion. Neurosci Lett.

